# MIF as an oncogenic driver of low‐heterogeneity melanomas

**DOI:** 10.1002/1878-0261.70031

**Published:** 2025-03-25

**Authors:** Thuy T. Tran, Gabriela Athziri Sánchez‐Zuno, Rajan P. Kulkarni, Harriet M. Kluger, Richard Bucala

**Affiliations:** ^1^ Department of Internal Medicine Yale Cancer Center, Yale School of Medicine New Haven CT USA; ^2^ Section of Rheumatology, Allergy & Immunology, Department of Internal Medicine Yale School of Medicine New Haven CT USA; ^3^ Department of Dermatology Oregon Health & Science University Portland OR USA

**Keywords:** D‐dopachrome tautomerase, immune resistance, macrophage migration inhibitory factor, melanoma, tumor heterogeneity, tumor‐associated macrophage

## Abstract

Identifying targets involved in tumor evolution and immune escape is an active area of research in oncology. Macrophage migration inhibitory factor (MIF) is an upstream immunoregulatory cytokine that promotes transformed cell proliferation and survival, and generates a tumor‐permissive immune landscape of immunosuppressive myeloid and T cells. Shvefel and colleagues have identified a key role for MIF in tumor progression in melanoma clones with low tumor heterogeneity. These findings provide important insights into the potential therapeutic utility of MIF antagonists and support ongoing research to utilize MIF pathway inhibitors for improved therapeutic outcomes.

## Graphical abstract

Shvefel and colleagues identified tumor‐secreted macrophage migration inhibitory factor (MIF) as an upregulated cytokine that mediates immune resistance in melanomas with low‐intratumoral heterogeneity. MIF and its functional paralogue D‐dopachrome tautomerase (D‐DT or MIF‐2) have overlapping but nonidentical signaling functions and are hypothesized to generate immune suppressive tumor microenvironments characterized by T‐cell suppression and tumor‐permissive macrophage populations.

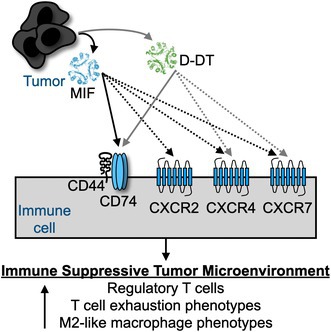

**Abbreviations**
D‐DT: D‐dopachrome tautomerase
KO: knockout
MDSC: myeloid‐derived suppressor cell
MIF: Macrophage migration inhibitory factor
TAM: tumor‐associated macrophage

Shvefel and colleagues have identified MIF as a prominent upregulated cytokine responsible for mediating immune resistance in melanoma in a detailed multiomics study. The authors used a novel animal model and an unbiased approach to evaluate nonrejected B2905 melanoma single‐cell tumor clones with low genotypic heterogeneity, which is associated with superior survival outcomes in human disease. Tumors were evaluated by a multimodal and longitudinal approach to identify candidate genes, pathways, and cells associated with growth and immune resistance. As expected for melanoma, nonrejected tumors had expanded populations of protumorigenic macrophages and exhausted T‐cell phenotypes. The authors then generated MIF knockout (KO) clones of nonrejected tumor cells and did not find any changes in proliferation *in vitro*, but MIF KO tumors grew more slowly in immunocompetent mice. Reconstitution of tumors with varying ratios of MIF wild‐type and MIF KO clones led to corresponding changes in tumor volume.

While several prior studies have supported a role for MIF in tumor growth and aggressiveness, there are little data on the complex and dynamic roles of how MIF signaling directly impacts immune cell function, including recruitment, proliferation, and signaling [1]. While MIF is a cytokine, it is important to appreciate that it has both signaling and intracellular functions that promote tumorigenesis. The authors’ conclusions dovetail with those of several recent studies that have used orthogonal approaches to identify MIF as a target of tumor aggressiveness and immune resistance. Tessaro *et al*. for example found similar results in a sarcoma model [2], and Liao *et al*. identified MIF as a regulator of anti‐inflammatory mononuclear cells in hepatocellular cancer models [3]. Collectively, these studies demonstrate that the tumor or tumor‐associated fibroblasts are the main contributors of MIF expression, and that MIF pathologically shapes the immune landscape to contribute to an immune suppressive tumor microenvironment. Given that MIF has been implicated as a mediator of macrophage heterogeneity, future studies classifying macrophages in the context of MIF elimination will expand our understanding of the effects of MIF in the tumor microenvironment. The impact of MIF loss or inhibition on tumor cells is also likely cell‐line and context‐dependent, as other tumor models have shown mixed results related to MIF's role in proliferation and tumor growth [4]. Complete inhibition of MIF signaling in immune cells, therefore, may have unexpected consequences in different tumor types given MIF's pleotropic effects on monocyte polarization, proliferation, antigen presentation, and T‐cell activation. Additional work also is needed to determine whether these findings are translatable to more aggressive tumors with high‐intratumoral heterogeneity.

Notably, far less is known about the second MIF family member D‐dopachrome tautomerase (D‐DT or MIF‐2). The two cytokines activate the MIF family cognate receptor CD74 and have a shared but not completely overlapping ability to activate the noncognate chemokine receptors CXCR2, CXCR4, and CXCR7 [5, 6] (Figure 1). Like MIF, D‐DT expression is elevated across multiple tumors where it is implicated in aggressiveness [7], and both mediators are highly correlated at the mRNA and protein levels in the tumor and blood of cancer patients, respectively [8, 9]. Ongoing studies by our group suggest that dual inhibition of MIF and D‐DT may be therapeutically superior in mouse models of immunoresponsive tumors when compared to single cytokine or CD74 inhibition [10].

**Fig. 1:**
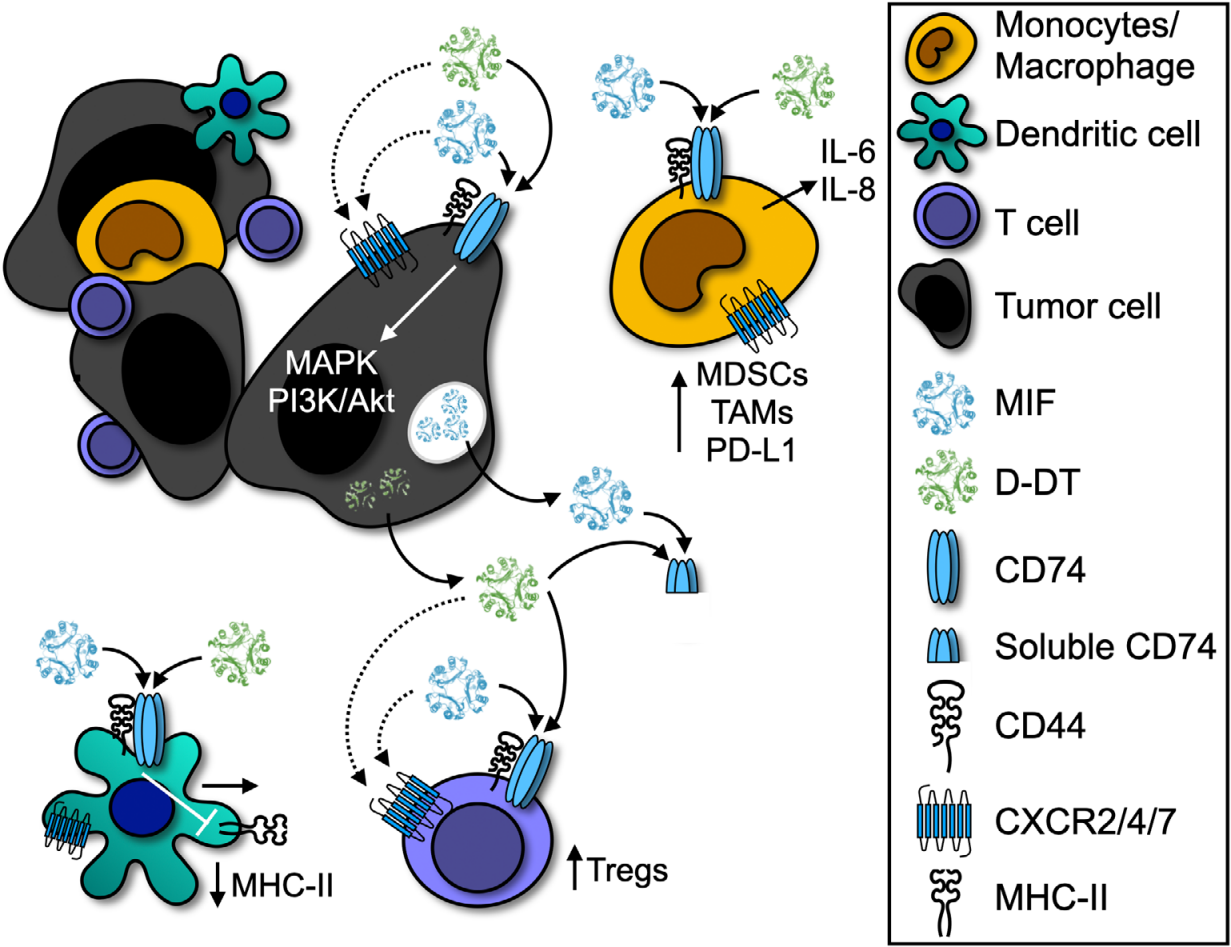
MIF and D‐DT signaling within the tumor microenvironment. Both macrophage migration inhibitory factor (MIF) and D‐dopachrome tautomerase (D‐DT) are produced by tumor and immune cells. Autocrine and paracrine signaling of MIF and D‐DT through binding of the cognate receptor CD74 and intracellular signaling via CD44 or noncognate receptors CXCR4/7 or CXCR2, in the case of MIF, activate pathways involved in cell proliferation and migration. Once secreted, additional MIF and D‐DT can bind receptors on monocytes/macrophages to recruit or polarize cells toward myeloid‐derived suppressor (MDSC) or tumor‐associated macrophage (TAM) populations, enhance IL‐6/‐8 production, and increase PD‐L1 expression to generate an immune suppressive tumor microenvironment. MIF and D‐DT also decrease MHC‐II on dendritic cells and increase regulatory T‐cell populations. CD74 can be cleaved into a soluble form that retains its ligand‐binding capabilities and may serve as a natural antagonist to MIF and D‐DT.

Thus, the study of MIF is complicated by the near ubiquitous expression of the cytokine and widespread presence of both its cognate and noncognate receptors across tumor and immune cells. Contrary to MIF and D‐DT being associated with poor survival, expression of their cognate receptor CD74 is associated with improved survival and increased intratumoral immune infiltration [9]. Furthermore, the extracellular domain of CD74 can be proteolytically cleaved to generate soluble CD74, which maintains ligand‐binding function and may serve as an antagonist. Elevated levels of soluble CD74 have been associated with improved survival in melanoma patients [11]. Similarly, Shvefel and colleagues found *Cd74* to be increased across several time points in the rejected clones and in response to MIF KO tumor clones in their scRNA‐seq analysis. Ongoing studies to evaluate CD74 expressing immune populations and their unique contribution in the tumor microenvironment will be informative, particularly in response to MIF‐pathway inhibition.

Therefore, the study by Shvefel and colleagues is consistent with studies that have demonstrated MIF to be a driver of immune evasion, tumor aggressiveness, and poor clinical outcomes in animal models, as well as in retrospective patient clinical data [9]. Notably, MIF expression in humans is regulated by polymorphisms in its promoter, namely a functional ‐794 CATT(5‐8) microsatellite and a nearby ‐173G/C SNP that occurs in linkage disequilibrium with the microsatellite. It remains unclear how this transcriptional regulation is associated with clinical outcomes, particularly because the microsatellite structural variant is not represented in SNP‐based genome‐wide association studies. This information becomes further noteworthy with inroads being made into MIF allele‐specific transcriptional inhibitors [12].

There is ongoing clinical interest in blocking the MIF signaling pathway, which has been demonstrated to be well‐tolerated [13, 14, 15]. Prior to further clinical investigation, it will be important to understand the direct impact of MIF targeting on immune cells to complement the existing data put forth by Shvefel and colleagues. The present data additionally supports consideration of expanding clinical studies of anti‐CD74 (milatuzumab), which are currently limited to B‐cell malignancies [13].

Further advances in the new era of immune therapy will likely involve combinations of blocking pro‐tumorigenic cytokines such as MIF, increasing immune stimulatory cytokines to promote T‐cell survival and macrophage activation, and traditional immune checkpoint inhibitors. These types of combinations should help improve response rates and address treatment resistance in melanoma and other cancers.

## Conflict of interest

RB and TT are co‐inventors on Yale‐managed patents for MIF pathway inhibitors. RB also serves on the advisory board for OncoOne.

## Author contributions

TT wrote the commentary and made the figure. GSZ, RK, HK, and RB edited and provided expertise on the content.
